# Calpain Activity Is Essential in Skin Wound Healing and Contributes to Scar Formation

**DOI:** 10.1371/journal.pone.0037084

**Published:** 2012-05-16

**Authors:** Dany Nassar, Emmanuel Letavernier, Laurent Baud, Selim Aractingi, Kiarash Khosrotehrani

**Affiliations:** 1 Université Pierre et Marie Curie-Paris 6, UMRS938, Paris, France; 2 INSERM, U938, Saint-Antoine Research Centre, Paris, France; 3 INSERM, U702, Paris, France; 4 Université Pierre et Marie Curie-Paris 6, UMRS702, Paris, France; 5 AP HP, Tenon Hospital, Department of Physiology, Paris, France; 6 AP HP, Tenon Hospital, Department of Dermatology, Paris, France; 7 Experimental Dermatology Group, UQ Centre for Clinical Research, The University of Queensland, Brisbane, Queensland, Australia; University of Debrecen, Hungary

## Abstract

Wound healing is a multistep phenomenon that relies on complex interactions between various cell types. Calpains are ubiquitously expressed proteases regulating several processes including cellular adhesion and motility as well as inflammation and angiogenesis. Calpains can be targeted by inhibitors, and their inhibition was shown to reduce organ damage in various disease models. We aimed to assess the role of calpains in skin healing and the potential benefit of calpain inhibition on scar formation. We used a pertinent model where calpain activity is inhibited only in lesional organs, namely transgenic mice overexpressing calpastatin (CPST), a specific natural calpain inhibitor. CPST mice showed a striking delay in wound healing particularly in the initial steps compared to wild types (WT). CPST wounds displayed reduced proliferation in the epidermis and delayed re-epithelization. Granulation tissue formation was impaired in CPST mice, with a reduction in CD45+ leukocyte infiltrate and in CD31+ blood vessel density. Interestingly, wounds on WT skin grafted on CPST mice (WT/CPST) showed a similar delayed healing with reduced angiogenesis and inflammation compared to wounds on WT/WT mice demonstrating the implication of calpain activity in distant extra-cutaneous cells during wound healing. CPST wounds showed a reduction in alpha-smooth muscle actin (αSMA) expressing myofibroblasts as well as αSMA RNA expression suggesting a defect in granulation tissue contraction. At later stages of skin healing, calpain inhibition proved beneficial by reducing collagen production and wound fibrosis. *In vitro*, human fibroblasts exposed to calpeptin, a pan-calpain inhibitor, showed reduced collagen synthesis, impaired TGFβ-induced differentiation into αSMA-expressing myofibroblasts, and were less efficient in a collagen gel contraction assay. In conclusion, calpains are major players in granulation tissue formation. In view of their specific effects on fibroblasts a late inhibition of calpains should be considered for scar reduction.

## Introduction

Skin wound healing is a multistep process that requires a close interaction of multiple cell types in a coordinate and orderly manner. In order to fill the defect, keratinocytes and fibroblasts proliferate and migrate into the center of the wound. Besides, cells from blood and bone marrow migrate into the wound and are implicated in inflammation and angiogenesis [1], [2], [3], [4], [5], [6]. All these phenomena require integrity in fundamental processes such as cell proliferation, motility and interaction with extracellular matrix. In addition, wound healing crucially relies on the mechanical activity of fibroblasts responding to TGFβ1 and to forces transmitted across focal adhesions formed essentially around integrins and their signaling pathways [7]. Although wound healing leads to the regeneration of normal skin in embryos and fetuses, it results after birth in visible scarring that is featured by excess collagen production and the absence of adnexa [8], [9].

Calpains are intracellular calcium-dependent cystein proteases. The major isoforms μ-calpain (calpain-1) and m-calpain (calpain-2) are ubiquitously expressed [10], [11], [12]. Both μ and m-calpain are heterodimers formed of a large subunit (80 kDa) specific for each isoform and an identical small subunit (28 kDa) named calpain 4 [13]. The activities of μ and m-calpains are redundant [14] and specifically regulated by calpastatin, a specific endogenous inhibitor [13]. Calpain activity enhances cell migration via proteolysis of tallin and Focal Adhesion Kinase (FAK), disassembly of focal adhesion complexes [15], [16], [17], reorganization of the cytoskeleton and stabilization of cellular protrusions [18], [19]. Calpain activity is required for induced keratinocyte motility via disassembly of adhesion plaques in response to epidermal growth factor (EGF) and Interferon-inducible protein 9 (IP-9, also known as CXCL11) [10]. Calpains have also been reported as modulators of multiple pathways of inflammation including the NF-κB signaling pathway via degradation of IκBα [20], migration and chemotaxis of neutrophils and monocytes via modulation of cell-to-matrix adhesion [21], [22]. Calpain activity is also required for angiogenesis and endothelial cell modulation of adhesion and motility in response to VEGF [23], [24], [25]. Although few processes have been shown to be specifically affected by one of the two ubiquitous calpains (μ or m) [10], [26], all *in vivo* studies were performed using inhibition of both isoforms. In fact, the activities of calpains μ or m are redundant [14] possibly due to their high sequence similarity and the shared calpain 4 sub-unit. They target the same substrates and have a common natural inhibitor and no specific inhibitor [13]. Therefore, in the literature as in this article calpain activity refers to the activity of both μ and m-calpains [27]. Interestingly, inhibiting calpain activity proved to be beneficial and reduced tissue damage in experimental models of inflammatory diseases [28], [29], cancer progression (reviewed in [27]), neurodegenerative [30] and cardiovascular diseases [31].

In the skin, μ-calpain is involved in the catabolism of filaggrin and filaggrin-2 during terminal differentiation of keratinocytes [32], [33], and the expression of both calpain μ and m is increased during skin wound healing [34]. In this study, we used calpastatin transgenic mice (CPST). This is a pertinent model since calpain activity is inhibited only in damaged organs. It allowed us to precisely evaluate how calpain inhibition affects skin wound healing *in vivo* and whether calpains can represent a putative therapeutic target to limit tissue damage and scar formation.

## Materials and Methods

### Mice

8 weeks-old Calpastatin transgenic female mice (CPST) on C57BL/6 background were used in these experiments [29], [31]. Age-matched wild-type C57BL/6 female mice (WT) were purchased from Janvier laboratories and used as controls.

### Surgical wound healing assay

Mice were anesthetized by inhalation of isofluorane (Aerane®, Baxter, Deerfield, IL). After depilation, 5 mm surgical wounds were generated. All tissues above the panniculus carnosus were excised. Wounds were performed on telogen stage skin, identified by the absence of pigment. Wounds were left uncovered until they were harvested.

Standardized pictures of the wounds were taken using a Sony Cybershot® 10.1 megapixels DSC-W180 digital camera. Wound surfaces at each time-point were expressed as percentages of the initial surface of each wound at day 0.

### Skin graft experiment

After depilation, a 2-cm2 flap from the back skin was excised and was then transplanted on recipient mice. Wounds were performed on grafts 2 months after transplantation.

### Microscopy, scoring and measurements

Five-micrometer cryosections were obtained. After permeabilization with Triton X-100, sections were blocked using 20% normal goat serum (DakoCytomation, Carpenteria, CA). Primary antibodies used included purified rat anti-mouse CD45 (1∶10; BD Biosciences, Le Pont de Claix, France), rabbit anti-mouse Ki67 (1∶200; Abcam Inc., Cambridge, MA), rabbit anti-Keratin 14 (1∶500, Covance, Denver, PA), rat anti-Mouse CD31 (1∶40; BD Biosciences), rabbit anti-Mouse Lyve-1 (1∶200; Abcam) and rabbit anti-Mouse alpha-Smooth Muscle Actin (αSMA) (1/200; Abcam). The secondary antibodies used were goat anti-rabbit IgG labeled with FITC, goat anti-rabbit IgG labeled with Texas Red, and goat anti-rat IgG labeled with Texas red (1∶100; Jackson ImmunoResearch, West Grove, PA). Slides were then counterstained with 0.3 µg/ml DAPI (Sigma-Aldrich, Lyon, France).

We used Nikon Eclipse 90i fluorescent microscope equipped with Nikon DS-Fi1C digital camera. For cell scoring, photographs of 3 different fields in the wound bed were taken and then labeled cells were counted and reported as percentage of total DAPI stained nuclei. Mean percentage of labeled cells was calculated for each specimen. For epithelialization measurements, diametrical sections were stained for keratin 14. The length of the newly formed epidermis was calculated by adding together the lengths of neo-epithelial tongues on both sides of the wound from the tip of the epidermis to the site of the first hair follicle at the wound margin. For vascular morphometric analysis, lymphatic and blood vessel count and surface were measured on 3 different fields on double-labeled section for CD31 and Lyve-1 as previously described [35]. Cell counts of CD45+ and αSMA+ cells in the granulation tissue were performed on 3 different fields on stained sections. Proliferating cells were assessed separately in the granulation tissue and at the epidermal wound edges. Fibrosis was quantified by measuring the surface of Sirius Red staining in the granulation tissue on diametrical sections of the wounds at day 10. The first hair follicles located deep in the dermis at both edges of the wounds were considered as the external limits of the granulation tissue. Measurements were done using ImageJ software (NIH).

### RNA extraction and quantitative PCR

Total RNA was extracted from *in vitro* differentiated fibroblasts and from skin wounds using the RNeasy total RNA Mini kit (Qiagen, Courtaboeuf, France). Total RNA (1 µg) was reverse transcribed using high capacity cDNA reverse transcription kit (Applied Biosystems, Courtaboeuf, France). Real-time PCR was conducted using SYBR®GREEN PCR Master Mix (Applied Biosystems) on ABI Prism 7300 (Applied Biosystems). mRNA values were normalized to the expression level of HPRT (Hypoxanthine-Guanine-Phospho-Ribosyl-Transferase) in human MRC-5 fibroblasts or RPS3 (ribosomal protein S3) in murine skin wounds. Each sample was analyzed in duplicate. Primer sequences were (Forward and Reverse; 5′-3′): CCAGCCGCAAAGAGTCTACA and TCAAGCATACCTCGGGTTTC for mCOL1A1, TTTGTGCAAAGTGGAACCTG and CGCAAAGGACAGATCCTGA for mCOL3A1, CTCCCTGGAGAAGAGCTACG and ATAGGTGGTTTCGTGGATGC for mA-SMA, ATCAGAGAGTTGACCGCAGTTG and AATGAACCGAAGCACACCATAG for mRPS3, ATGTTCAGCTTTGTGGACCTC and CTGTACGCAGGTGATTGGTG for hCOL1A1, CTGTTCCAGCCATCCTTCAT and TCATGATGCTGTTGTAGGTGGT for hA-SMA, GGACCTCCTGGTGCTATAGGT and CGGGTCTACCTGATTCTCCAT for hCOL3A1, AGTTGAGAGATCATCTCCAC and TTGCTGACCTGCTGGATTAC for hHPRT.

### Fibroblast differentiation assay

Confluent cultures of Human MRC-5 fibroblast line were serum-starved for 48 hours, incubated with Calpeptin (20 or 40 µM) or vehicle (DMSO), then treated with 10 ng/ml TGF-β1 (PeproTech, Rocky Hill, NJ) or Phosphate-Buffered Saline (PBS) vehicle for 72 hours [36].

### Collagen gel contraction

Analysis of floating collagen gel contraction was performed as previously described [36], [37]. Ice-cold Collagen I Rat Tail (Invitrogen, Paisley) was diluted at 30% v/v in serum free culture medium and then mixed with fibroblasts (10^5^ cells/ml) and 10 ng/ml TGF-β1, with Calpeptin (40 µM) (Calbiochem, Merck KGaA, Darmstadt, Germany) or vehicle (DMSO). Changes in surface area were measured every 24 hours for 5 days.

### Statistical analysis

Data were expressed as means ± SEM. Means between 2 groups were compared using the Mann Whitney test. Differences between multiple groups were analyzed by 1-way ANOVA. P<0.05 was considered to be statistically significant.

## Results

### Wound healing and re-epithelialization are delayed in CPST mice

**Figure 1:**
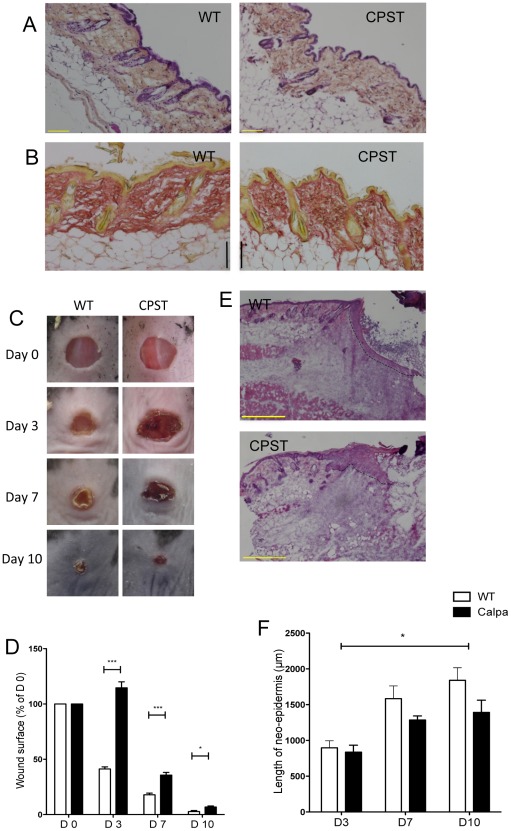
Delayed wound healing in CPST mice. Normal skin sections of CPST and WT mice stained with Hematoxylin-Eosin-Safran (A) and Sirius Red (B) do not show any differences at steady state. 5 mm punch biopsies were performed on CPST and WT mice. Photographs (C) and surface (D) of the wounds at multiple time points showing delayed wound closure in CPST mice. Diametrical sections of wounds stained with Hematoxylin-Eosin depicting neo-epidermis (dashed lines) (E) and length of neo-epidermis on keratin 14-labeled sections (F) showing delayed reepidermization in CPST mice. n = 7 per group per time point. Scale bars: (A and B) 100 µm, (E) 500 µm. (D) Mann-Whitney test, (F) ANOVA, *P<0.05; ***P<0.001. (D and F) mean +/−SEM.

To address our hypothesis that calpain activity is essential during skin wound healing, we have used well-characterized transgenic mice that overexpress calpastatin, a natural and specific inhibitor of both μ and m-calpains. Homozygous calpastatin transgenic mice (CPST) do not show a skin phenotype at steady state. Morphology of the epidermis and dermis were in accordance similar to WT controls (**figure 1A and B**). We performed 5 mm excisional wounds on the back of CPST and WT mice and monitored wound closure. Healing was strikingly delayed in CPST wounds particularly at the initial stages (mean wound surface on day 3: CPST vs WT = 115% vs 41%, p<0.0001, ANOVA) (**Figure 1** **C and D**).

**Figure 2:**
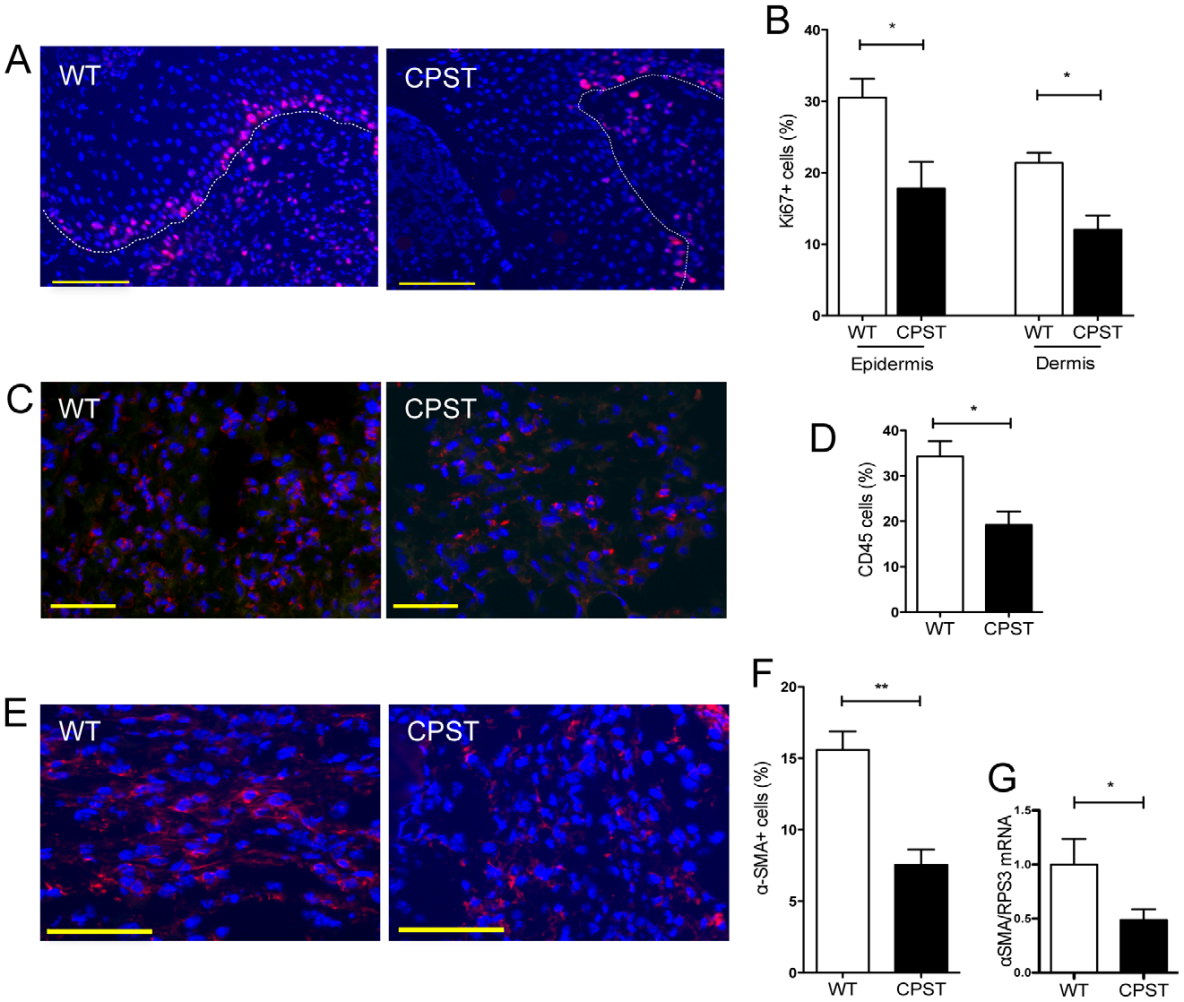
Impaired proliferation and granulation tissue formation in CPST wounds. Ki67 staining (red) on CPST and WT wound sections at day 7 (A) showed reduced numbers of proliferating cells in the epidermal wound edges and the granulation tissue dermal cells (B). CD45 staining (red) and counts (C and D) on day 3 wound sections showed reduced numbers of inflammatory cells in CPST wound beds (D). α-Smooth-Muscle-Actin staining (αSMA) (red) and counts (E and F) showed reduced numbers of myofibroblasts in CPST wounds. mRNA levels of αSMA in day 3 CPST and WT wounds. Representative photos of wound edges are depicted in A; epidermis is delineated in dashed lines. Representative photos of wound granulation tissue are depicted in (C) and (E). Nuclei were stained with DAPI (blue). n = 7 per group. Scale bars: (A) 100 µm, (C and E) 50 µm. Mann-Whitney test, *P<0.05, **P<0.01. (B, D, F and G) mean +/−SEM.

Analysis of the wound specimens enabled us to decipher more precisely the cellular mechanisms in this healing delay. We first assessed the re-epithelialization by measuring the length of the newly formed epidermis covering the granulation tissue at different time points. Neo-epidermis was significantly shorter in CPST mice as compared to WT (**Figure 1E and F**). This reduction was accompanied by a lower number of Ki67 positive proliferating keratinocytes in the CPST wound edges on day 7 (**Figure 2A and B**).

### Impaired granulation tissue formation in CPST mice

**Figure 3:**
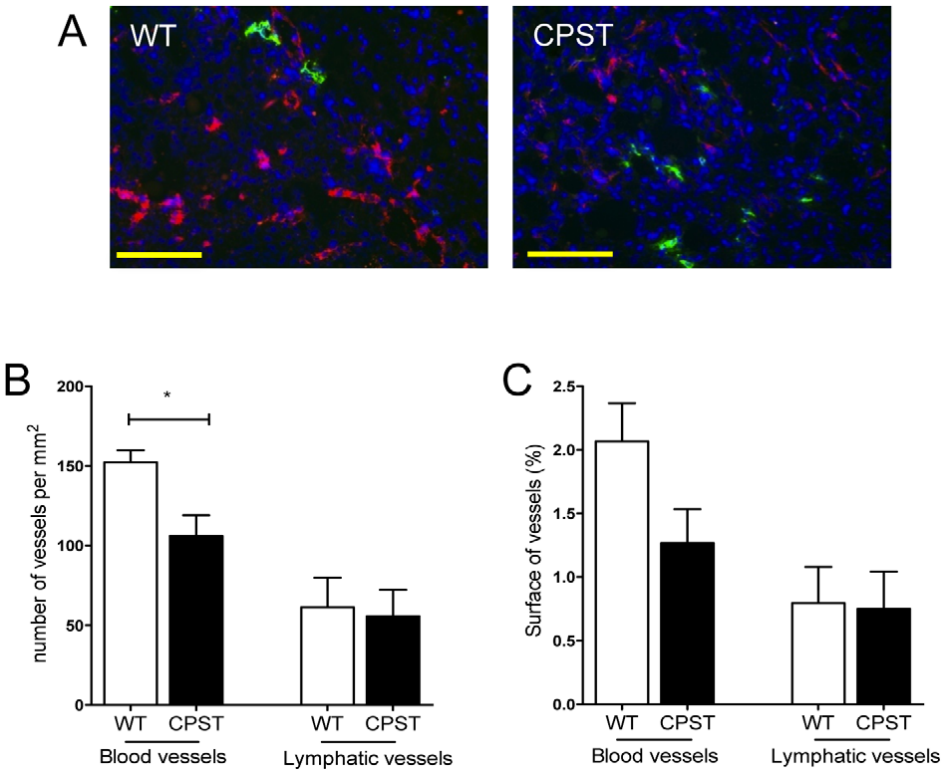
Impaired angiogenesis in CPST wounds. Wound sections (day 7) of CPST and WT mice were double-labeled for CD31 (red) and Lyve1 (green). Nuclei were stained with DAPI (blue). Representative photos of wound granulation tissue in WT and CPST mice are depicted in (A). Number (B) and surface (C) of blood and lymphatic vessels in the wound beds. n = 7 per group. Scale bar (A) 100 µm. Mann-Whitney test, *P<0.05. (B and C) mean +/−SEM.

We also observed reduced numbers of Ki67+ proliferating cells in the wound beds of CPST mice compared to WT (**Figure 2B**) indicating an alteration of granulation tissue formation. The number of inflammatory CD45+ cells was significantly reduced in CPST wounds (CPST vs WT, 19% vs 34% of total cells, p<0.05, Mann-Whitney) (**Figure 2C and D**). There was also a lower number of αSMA+ contractile myofibroblasts in CPST wounds (CPST vs WT, 8% vs 16% of total cells, p<0.01, Mann-Whitney) (**Figure 2E and F**). This was in accordance with a lower αSMA mRNA levels in CPST wounds measured on the same time point (**Figure 2G**). Finally, quantitative assessment of blood and lymphatic angiogenesis was done on wound sections double-labeled for CD31 and Lyve1: CPST wounds showed a significant decrease in blood vessel density and an important, although not significant, decrease in blood vessel surface when compared to controls (**Figure 3A–C**).

### Impaired cell recruitment in the wound beds of CPST mice

**Figure 4:**
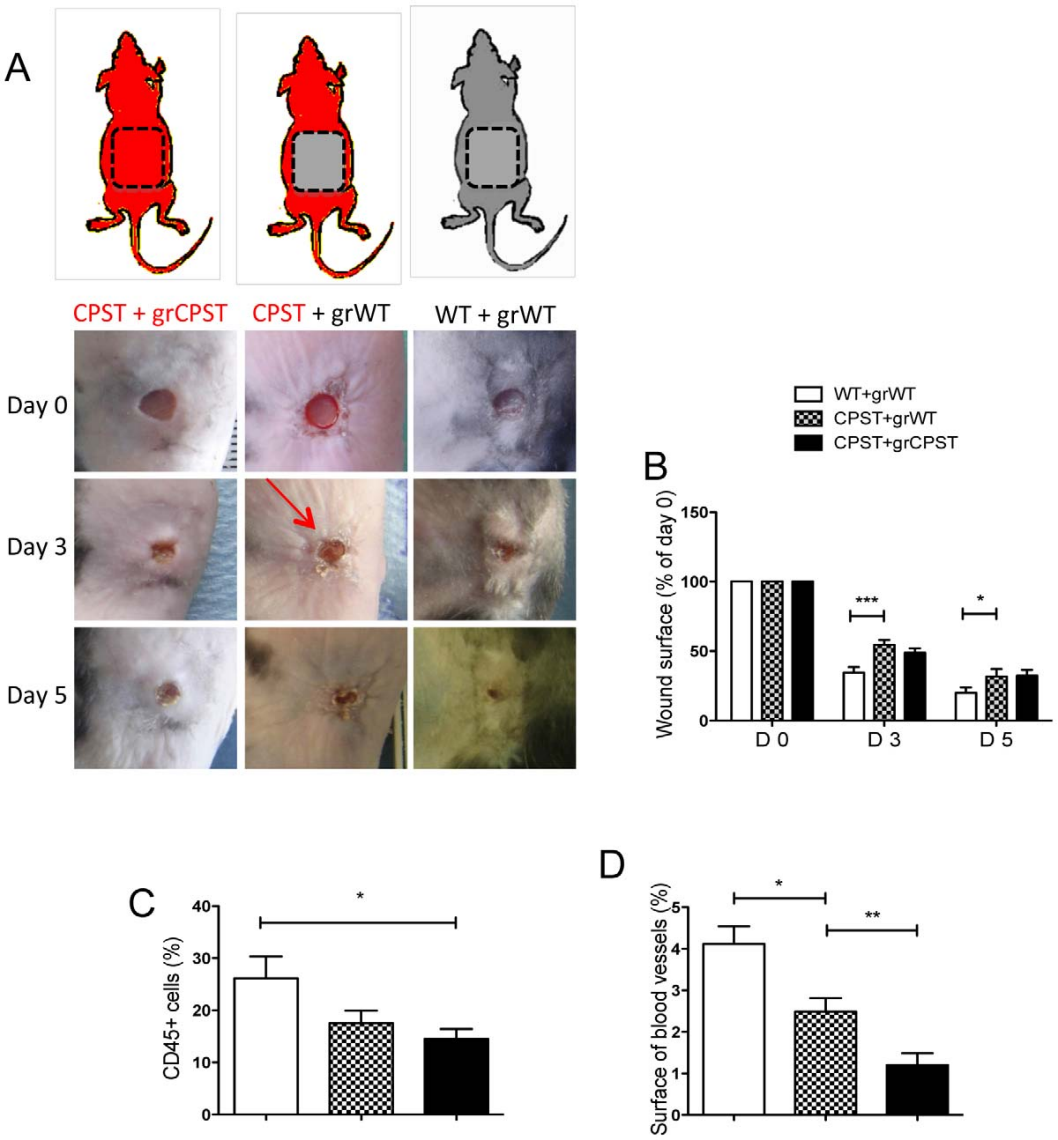
Impaired cell recruitment in the wound beds of CPST mice. WT Skin flaps were transplanted on the back of CPST recipients (CPST+grWT) with corresponding controls (a). 2 months after transplantation, excisional biopsies were performed on grafts and monitored over time. Photographs (A) and surface (B) of the wounds at different time points. Number of CD45 positive cells (C) and surface of CD31+ blood vessels (D) were evaluated. n = 6 per group. Mann-Whitney test, *P<0.05; **P<0.01. (B–D) mean +/−SEM.

Wound healing leads to recruitment of cells from peripheral circulation or marrow [1], [2]. Therefore, we addressed the contribution of local versus systemic calpain inhibition in the observed delay in wound healing in CPST mice. Skin flaps were transplanted from WT mice on the back of CPST recipient mice (WT/CPST). Controls were CPST skin transplanted on CPST mice (CPST/CPST) and WT skin on WT mice (WT/WT). Two months after transplantation, 4 mm excisional wounds were performed on the grafts and the healing was monitored. As expected, CPST/CPST wounds healed significantly slower than WT/WT ones. Interestingly, there was also a significant delay in closure of WT/CPST wounds when compared with WT/WT wounds (**Figure 4A and B**). This suggested that the systemic calpain inhibition in CPST recipients affected healing in CPST mice, rather than a defect in CPST skin cells. On the other side, WT/CPST wounds did not heal faster than the CPST/CPST wounds also indicating that the grafted skin components, especially the epidermis, were not directly involved in the delayed healing observed in CPST wounds. *In situ* analysis of the wounds showed a decrease in the inflammatory cell infiltrate in WT/CPST wounds as compared to WT/WT wounds (**Figure 4C**). There was also a significant decrease of blood vessel surface in WT/CPST compared to WT/WT wounds. Interestingly, CPST/CPST wound beds showed a significant decrease in wound angiogenesis compared to WT/CPST specimens (**Figure 4D**) suggesting that both local and distant cell mobilization and/or proliferation –that participate in wound inflammation and angiogenesis– were calpain dependent.

### Calpain inhibition reduces collagen deposition in wound beds and inhibits myofibroblast differentiation

**Figure 5:**
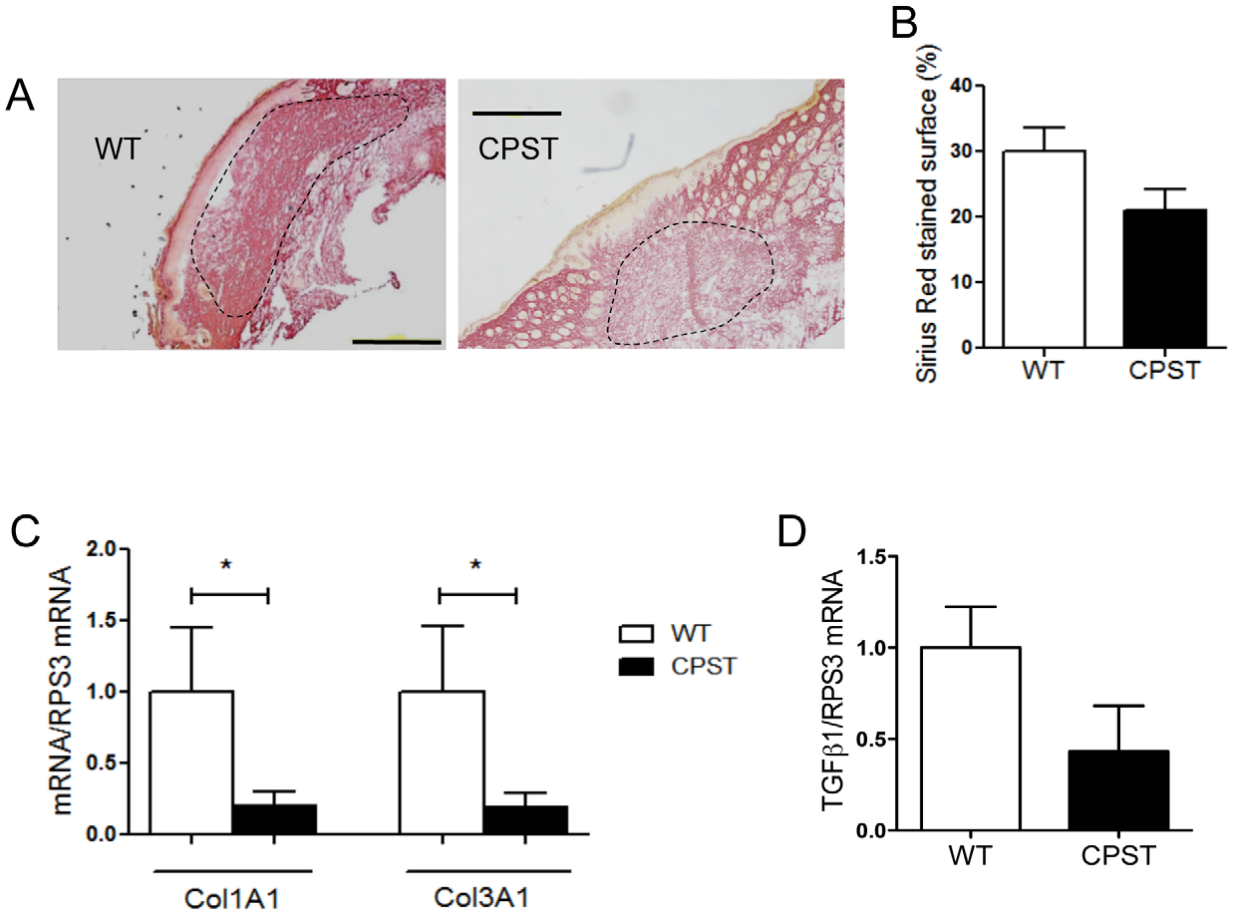
Calpain inhibition reduces scar formation in skin wounds. Day 10 Wounds of CPST mice show less Sirius Red staining and more epidermal adnexa regeneration compared to WT mice (A). Relative surface of Sirius Red staining in WT and CPST wounds on day 10 (B). mRNA levels of Collagen1A1 and TGFβ1 in day 3 CPST and WT wound (C and D). (A–D) n = 7 per group. Scale bars: (A) 500 µm. Mann-Whitney test, *P<0.05. (B–D) mean +/−SEM.

**Figure 6:**
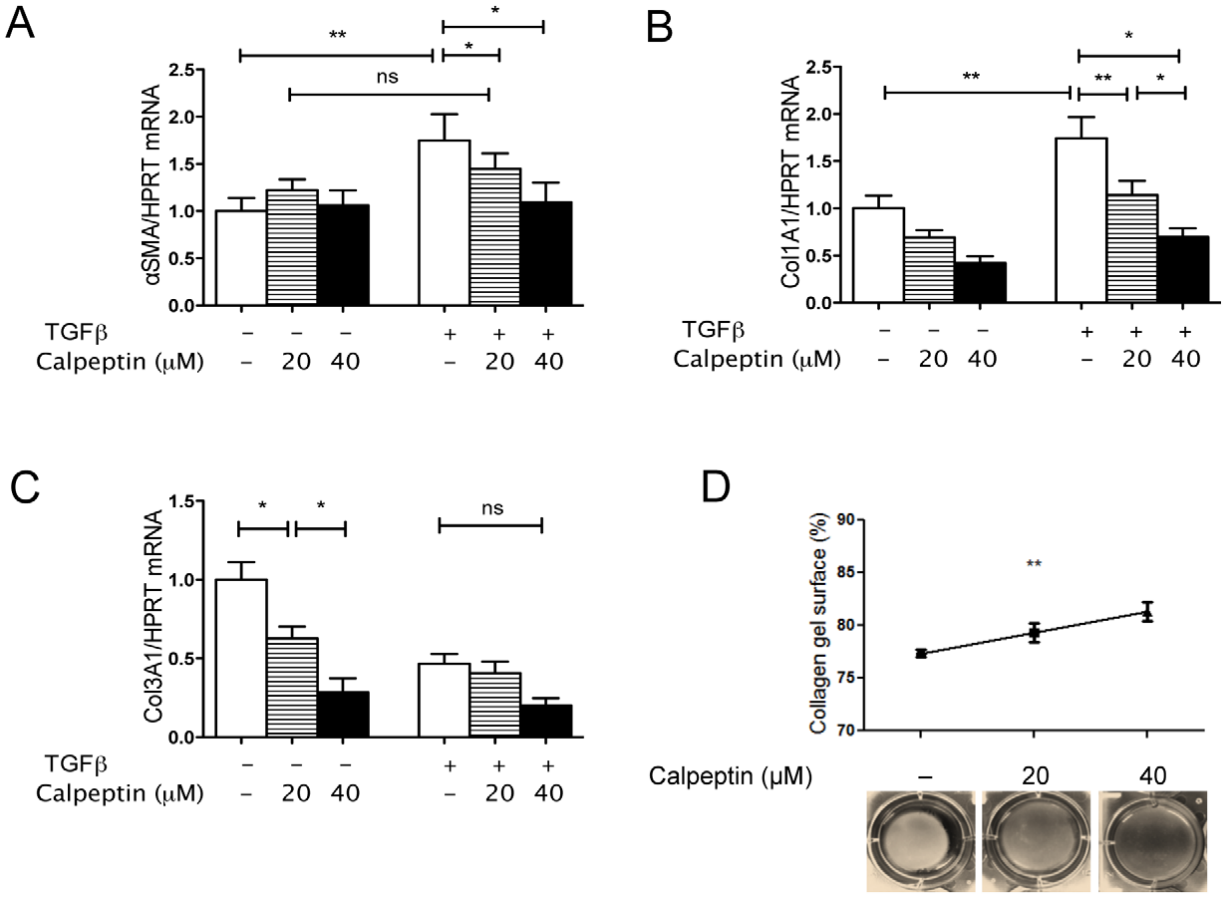
Calpain inhibition inhibits myofibroblast differentiation, contraction and collagen synthesis. MRC-5 fibroblasts were stimulated with TGFβ1 with or without addition of Calpeptin. mRNA levels of αSMA (A), Collagen1A1 (B) and Collagen3A1 (C) after TGFβ1 stimulation. MRC-5 cells embedded in floating collagen gels were stimulated with TGFβ1. Photos (D) of collagen gels with or without Calpain inhibition with Calpeptin. (A–D) n = 4. (A–C) Mann-Whitney test; (D) ANOVA; *P<0.05; **P<0.01. (A–D) mean +/−SEM.

The decrease in αSMA+ myofibroblasts in CPST wounds prompted us to assess the contractile function of skin fibroblasts and collagen deposition in CPST mice. On day 10, Sirius Red staining showed a tendency towards reduced fibrosis in CPST wounds (**Figure 5A and B**). Accordingly, collagen1A1 and collagen3A1 mRNA levels (day 3) were significantly lower in CPST than in WT wounds (**Figure 5C**). There was also a trend towards a decrease in TGFβ1 mRNA levels in CPST wounds (**Figure 5D**). TGFβ is known to induce fibroblast differentiation into contractile myofibroblasts and to stimulate the synthesis of type I collagen that replaces the early type III collagen [38], [39]. To analyze the role of calpains on fibroblast response to TGFβ, we stimulated human MRC-5 fibroblasts with TGFβ in the presence of calpeptin, a pan-calpain inhibitor. On qPCR, calpeptin significantly inhibited TGFβ-induced αSMA RNA levels (**Figure 6A**). Calpain inhibition also reduced collagen I expression by fibroblasts upon TGFβ stimulation and Collagen III transcription at steady state (**Figure 6B and C**). Moreover, calpeptin inhibited TGFβ-stimulated collagen gel contraction in a dose-dependent manner showing that calpain inhibition alters the fibroblast/myofibroblast contractile properties (**Figure 6D**).

## Discussion

This study shows that the *in vivo* inhibition of calpains does not alter the overall structure of the normal skin while it deeply modifies wound healing by affecting all the steps of this process. In mice overexpressing calpastatin, a physiological inhibitor of calpains, the healing was strikingly delayed mainly in the early stages. The proliferation of keratinocytes and granulation tissue cells, the density of the inflammatory infiltrate and of blood vessels were reduced in CPST mice. At a later stage, scar formation, collagen production and both collagens I and III transcription were reduced in CPST wounds. This was in accordance with *in vitro* data showing that calpain inhibition in a human fibroblast cell line led to a reduction of collagen I synthesis, myofibroblast differentiation and contractile function.

Calpastatin inhibits both μ and m-calpains. Thus, the impaired wound healing observed in CPST mice cannot be strictly attributed to one of the two isoforms. However, several *in vitro* studies showed that there is a redundancy in the cellular functions of μ and m-calpain. In a murine embryonic fibroblast line, only a knock down of the shared sub-unit calpain 4 –and not of μ or m-calpain alone– inhibits the contractile and adhesive properties of the cells [14]. Moreover, mice bearing a constitutional knockout of μ-calpain do not display an overt phenotype except for a moderate platelet dysfunction [40]. Calpastatin transgenic mice used in this work are a reference model to study the role of calpain activity in the pathophysiology of organ damage. In fact, in these mice, calpain activity is not reduced under steady state conditions while it is inhibited by the transgene only in damaged tissues [41], [42]. Since μ and m-calpains are both up-regulated in skin wounds and since calpain activity is only inhibited under pathologic conditions in CPST mice, our model appeared as a pertinent one to assess calpain activity implication in wound healing.

Calpain activity appeared essential in the establishment of the inflammatory phase of wound healing since the CD45+ cells were significantly reduced in mice overexpressing calpastatin. Since both μ and m-calpains display two peaks occurring at days 1 and 5 of the wound healing [34], the data we show here suggests that the first peak corresponds to the early inflammatory step in healing [34]. During this stage, the wound bed is infiltrated with a high number of neutrophils and macrophages [4], [5], [43]. These derive from the recruitment of circulating inflammatory cells as well as the recruitment of hematopoietic progenitor cells from the bone marrow [1], [44]. The graft experimentations that we performed showed a reduction in CD45+ cells in wounds on WT skin grafted on CPST recipients. These data indicate that the calpain activity in the distant niche of inflammatory cells *per se* is important for their recruitment in wound beds independently of the skin background. In fact, in human differentiated neutrophils as well as in monocytes, calpain inhibition was shown to increase migration and chemotaxis *in vitro* [45], [46]. On the other hand, calpain inhibition reduces the TNFα-induced adhesion of neutrophils and promotes random migration, suggesting that calpains mediate the arrest signal of these cells at sites of inflammation [22]. Our results as well as others in experimental glomerulopathy [29], pleurisy or arthritis [28] confirm *in vivo* the importance of calpains in the build-up of inflammation potentially through this mechanism.

Calpastatin transgenic mice displayed reduced angiogenesis in their wound beds. The wounds on WT skin transplanted on CPST recipients showed decreased vessel surface as compared to WT grafts on WT recipients. Interestingly, angiogenesis in wounds on CPST skin grafted on CSPT recipients was also significantly reduced as compared to WT grafts on CPST recipients. It is well known that recruited monocytes/macrophages play a key role in promoting skin wound angiogenesis [5]. On the other hand vessel formation in the wound bed results from the budding of dermal endothelial cells; while the contribution of circulating endothelial progenitors has recently been questioned [47]. Our graft experimentations indicate that local but also distant cells or progenitors were implicated in wound angiogenesis and that both were affected by calpain inhibition. All these results are in accordance with the increase of m-calpain expression and activity found in endothelial cells after VEGFa stimulation. Calpain activity promotes angiogenesis through enhanced cell detachment from matrix and reorganization of cytoskeleton [23], [25], [48].

Calpain inhibition was beneficial in late stages of wound healing featured by a reduction in collagen I, αSMA and TGFβ expression and scar formation. This may correspond to the second peak of calpain activation after skin wounding [34]. The link between inflammation and fibrosis is very tight in skin wound healing [49] as well as in other tissue injuries as in myocardial fibrosis secondary to hypertension [50]. Accordingly, it has recently been shown that collagen deposition and scarring during wound healing can be affected by early macrophage depletion [5]. Macrophages are a major source of TGFβ during wound healing [51], [52]. Thus, the decrease in wound fibrosis observed in CPST mice could be –at least partly– caused by a reduced inflammatory cell infiltrate in the granulation tissue. We also observed a decrease in TGFβ expression in CPST wounds. In a previous work, two of us (E.L. and L.B.) showed –using the same transgenic mice– that calpastatin overexpression limits hypertension-induced intimal fibrosis in blood vessels via a decrease in inflammatory cell recruitment and secretion of MIP-1 [31]. These data are also in line with multiple studies showing that, despite the pleiotropic effect of calpains in basic cell functions, inhibiting calpain activity limits tissue damage in target organs [28], [29], [42].

Finally, our results clearly show that calpain inhibition has direct effects on fibroblasts regardless of inflammation. Indeed, expression of collagen I and III by fibroblasts was inhibited by calpeptin, a calpain inhibitor. Morevover, calpeptin significantly inhibited TGF-β induced collagen I overexpression. The *in vivo* microenvironment influences collagen production by fibroblasts through a large spectrum of molecules, TGFβ being classically the most important. Beside collagen deposition, wound contraction participates to scar formation in later stages of wound healing [38], [53], [54]. Skin wounds of CPST mice showed lower numbers of αSMA positive myofibroblasts in wound sections and lower αSMA mRNA levels in wound extracts. Pharmacologic inhibition of calpain activity by calpeptin limits fibroblast differentiation into αSMA positive myofibroblasts *in vitro*, and limits their contractile properties. These results are in accordance with previous models of bleomycin-induced lung fibrosis where collagen1A1 synthesis in lung fibroblasts was also inhibited by calpastatin [55].

In conclusion, our study sheds new light on the role of calpains in wound healing. Although calpain inhibition of inflammatory cell recruitment and angiogenesis in the early stages of wound healing delays the healing, the reduction in scar formation observed at later stages has proven beneficial. The latter effect seems to be due to a direct effect on fibroblasts and their response to TGFβ. Calpains are target proteins that should therefore be considered for further studies in scar formation and skin fibrosis. Moreover, new calpain inhibitors are now developed and trials are conducted in clinical areas as Alzheimer's disease and cardiovascular disorders. Special attention should be paid to wound healing and ulcer development in these patients [56].
